# Ten quick tips for ensuring machine learning model validity

**DOI:** 10.1371/journal.pcbi.1012402

**Published:** 2024-09-19

**Authors:** Wilson Wen Bin Goh, Mohammad Neamul Kabir, Sehwan Yoo, Limsoon Wong

**Affiliations:** 1 Lee Kong Chian School of Medicine, Nanyang Technological University, Singapore, Singapore; 2 School of Biological Sciences, Nanyang Technological University, Singapore, Singapore; 3 Center for Biomedical Informatics, Nanyang Technological University, Singapore, Singapore; 4 Center of AI in Medicine, Nanyang Technological University, Singapore, Singapore; 5 Division of Neurology, Department of Brain Sciences, Faculty of Medicine, Imperial College London, London, United Kingdom; 6 School of Computing, National University of Singapore, Singapore, Singapore; 7 Yong Loo Lin School of Medicine, National University of Singapore, Singapore, Singapore; bioinformatics.ca, CANADA

## Abstract

Artificial Intelligence (AI) and Machine Learning (ML) models are increasingly deployed on biomedical and health data to shed insights on biological mechanism, predict disease outcomes, and support clinical decision-making. However, ensuring model validity is challenging. The 10 quick tips described here discuss useful practices on how to check AI/ML models from 2 perspectives—the user and the developer.

## Introduction

The rapid advancement of Machine Learning (ML) and Artificial Intelligence (AI) technologies has sparked a transformative revolution across diverse domains. The convergence of sophisticated algorithms, powerful computing capabilities, and an abundance of data has propelled these technologies to the forefront of innovation, significantly impacting fields such as biomedicine, health, and technology. The increasing importance of ML and AI can be attributed to their unparalleled ability to decipher complex patterns [1], extract valuable insights [2], and automate decision-making processes [3].

AI/ML models are increasingly deployed on biomedical and health data. These models can be used to shed insights on biological mechanisms, predict disease outcomes, and support clinical decision-making. We see some notable successes in, for example, protein structure prediction [4] and in clinical decision support [5], but there have also been challenges and less stellar outcomes. For example, in drug target prediction, IBM Watson did not live up to expectations in streamlining and accelerating the drug discovery process. And in meta-analysis, AI models were not able to yield quality explanations due to issues such as random feature substitutability [6–8] and the existence of many high-performing models in the Rashomon set [9–11].

**Fig 1:**
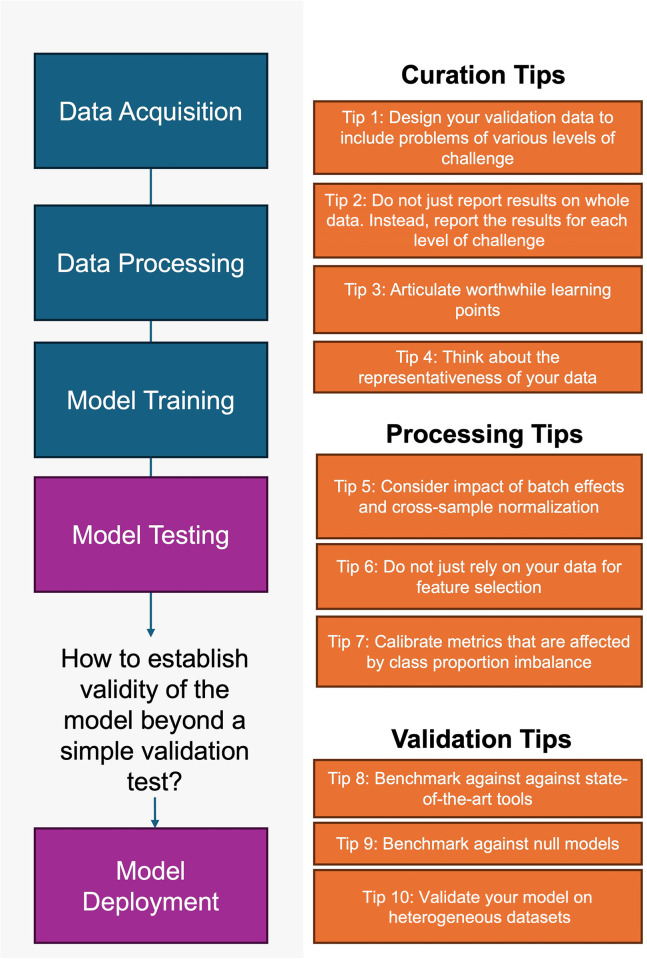
The 10 tips for establishing AI or ML model validity. Curation tips encompass considerations for designing both training and validation sets. Processing tips pertain to the methodology for feature selection, metric calibration, and batch correction/normalization of data. Validation tips focus on the methods used to assess the quality of learning exhibited by models.

AI and ML are, ultimately, tools. The effectiveness of these tools depends on how well the human user is capable of building and exploiting them [12]. Current literature provides general guidelines on using ML models in different areas like chemical science, COVID-19 data, etc. [13–16], which focuses more on input data, leakage, reproducibility, class imbalance, parameter tuning, and choosing an appropriate metric. Unfortunately, there is a notable absence of best practices concerning the creation of high-quality training datasets, the evaluation of trained models, and the calibration for interpreting model performance in real-world situations. A set of 10 quick tips is described here as an initial stride toward filling this gap (**Fig 1**). We explore the implications of these tips for 2 primary stakeholders: the developer and the user.

## Tip 1: Design your validation data to include sufficient problems of various levels of challenge

In AI/ML, we could treat the model as a student who was taught (trained on data). The validation step is therefore the equivalent of a quiz. But unlike education and summative assessment frameworks, there is no formal guidance on evaluating the “challenge” of the validation set. Indeed, people seldom check for this. Currently, people test a model by taking some data and testing the model with it. This plug-and-play approach displays a lack of deliberate effort in curating and designing validation data, which can lead to subjective and inaccurate assessment of a model’s performance.

Recently, we noticed an issue in AI/ML involving what we call “easy test sets.” An easy test set is validation data enriched for easy questions that lead toward inflation of AI performance. When validation data comprises mostly of “easy” problems, any poorly trained AI/ML model will do well.

Deep Neural Networks (DNNs), a type of AI, are increasingly used in protein function prediction. At first glance, they appear to be very successful. These include DeepFam (2018, CNN) [17], DeepGraphGO (2021, CNN + DNN, multimodal) [18], DeepGoZero (2022, zero-shot learning) [19], etc. However, what we found is that the good performance of some of these AI deep learning models has less to do with quality of learning or the sophistication of the model architecture, and more to do with easy problem inflation in the test set. A cutting edge DNN algorithm, DeepFam, claimed approximately 95% accuracy on its test sets. But when we inspected the test sets, we found that <5% of the test instances could be considered as “twilight zone” proteins—that is, proteins having less than 30% pairwise sequence identity to proteins in the training set [20]. Twilight zone proteins count as “challenging” problems for an AI trained to predict function based on protein sequence because they lack an obvious parallel in the training set. In Kabir and Wong, DeepFam was specifically evaluated on twilight zone proteins and the performance was disappointing, falling below 50%, a stark contrast to the reported 95% accuracy [20].

In protein function prediction, we can categorize problems into easy, moderate, or hard, based on their sequence similarity to proteins in the training set as a guideline. We can also model the proportions of these problems based on the distribution of sequence similarities found in real genomes to the reference protein database or the training set, so that the performance of the model would be a good proxy when evaluated on future new data. In cases where the model performs well on all difficulty levels, we can conclude that the model has learned meaningfully given that there is no leakage between training and validation data.

**Fig 2:**
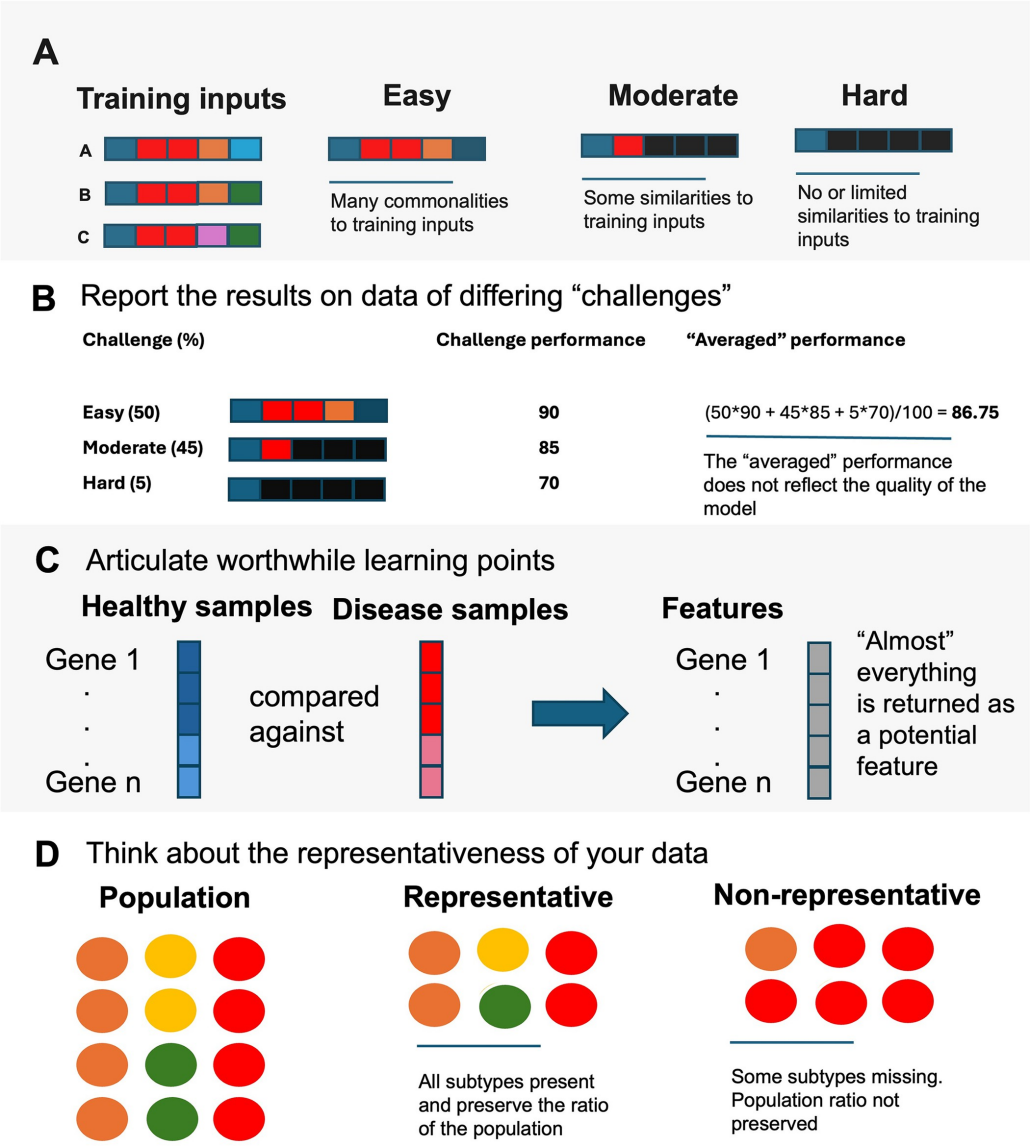
Curation tips covering tips 1 to 4.

When applying tip 1, it is important to identify what would constitute a challenge in the learning task. The challenge should be meaningfully and readily measured and broken down into different levels (**Fig 2A**). The developer should curate the validation set to contain enough samples from different levels of difficulties, to give insight on whether the model is performing well only on easy targets or on all levels of difficulties. For a user, before adopting a model to solve a problem, the model should be validated properly with a thorough test set according to tip 1. Comparing results for each difficulty level will help the user to identify a better model. Besides crafting problems of easy, moderate, and high challenge, a second layer of check is to minimize correlation or similarities with training data.

## Tip 2: Do not just report results on whole data. Instead, report the results for each level of challenge

Returning to our earlier example on protein function prediction, Kabir and Wong [20] showed that test sets predominantly comprise easy cases and do not illuminate whether the classifier would perform well on tough cases. This means the overall performance would be skewed by easy problems. This may not be such a big problem, if hard problems are rare. However, where protein sequence function prediction is concerned, hard problems stemming from twilight zone proteins can easily comprise 30% or more in a newly sequenced genome. For example, Lobb and colleagues [21] reported that on average across the bacterial tree of life, 48% of a bacterial genome could not be functionally annotated based on sequence similarity. And Bovio and colleagues [22] reported that conserved InterPro domains could not be identified in 40% of the proteins of a newly assembled *Periconia digitata* genome. So, there are more tough cases in real life. Simply providing an overall score on performance across problems of different levels of challenge does not help us understand whether the learner has really learnt.

By stratifying performance across easy, moderate, and hard problems based on protein sequence identities (**Fig 2B**), it becomes clear that some deep learning models like DeepFam are weak at solving hard problems.

To apply tip 2 for a developer, the validation datasets should be stratified by challenge levels, and the performance of the model should be reported separately for each challenge level. For example, test instances in a validation dataset might be stratified into various difficulty levels based on their similarity to the training set. Stratifying performance by challenge levels offers the developer a clear idea of how the model works for easy, moderate, and difficult samples, providing valuable information on the quality of learning for subsequent refinement of the model. For a user, tip 2 should be applied to check a model’s performance across different challenge strata of the user’s test set. In particular, the user needs to be satisfied with the model’s performance for the appropriate challenge level before adopting the model.

## Tip 3: Articulate worthwhile learning points

AI/ML techniques can learn subtle and complex signals from high-dimensional data, which, in turn, is useful when trying to train a classifier to discriminate class labels corresponding to different groups. However, the more similar the samples from different classes are, the harder it is for the AI/ML to discriminate.

If distinguishing classes that are very different is what the user wants, the developer will of course still develop the model as per the request of the user. It is likely the endeavor will be successful, and the AI/ML will perform well. However, it is also likely the AI/ML will pick up on superficial differences. On the other hand, training an AI/ML model to differentiate between 2 groups that are very similar to each other is more challenging than doing so for 2 groups that are highly dissimilar. When the groups are similar, there may be subtle differences that are difficult to capture, leading to a higher risk of misclassification.

Beyond model accuracy, if we want to articulate learning points from the outcome of learning, there should be some degree of training set curation that can help us arrive at better levels of explainability. Suppose we want to build a model for predicting cyclins from non-cyclins (see tip 4 for the real-life version of this example), besides including a list of proteins that are labeled cyclins and non-cyclins, we should also include in the test set non-cyclins, which are as similar to cyclins as possible (for example, other proteins involved in regulating cell cycles that share similar functions or structures with cyclins—CDK inhibitors, APC/C, Rb, and checkpoint kinases). That is, there might be instances that are very similar to each other, even though they are from different classes. The developer should make sure to include these for training and testing his model.

Doing this potentially allows deeper learning points to be made. For example, should the model tend to fail on closely similar samples of different classes, we can infer that the model has only learnt superficially. This is related to tip 2, since we can extrapolate that such closely similar samples could be instances of hard problems. However, we can do more. Given that there are different types of “sublabels” (for example, shared functions or structure with cyclins) associated with the non-cyclins, we can also evaluate if there are biases in performance associated with these sublabels. Perhaps, these “biases” may cause us to reevaluate the veracity or objectiveness of the initial class labels. We should not only look superficially at the AI/ML models overall performance but also use the construct of the class labels to deepen our understanding of the domain.

When a developer applies tip 3, it is crucial to consider what is being compared with (**Fig 2C**). If the classes are too different, many superficial features may arise, making it challenging to understand why the model performs well and which features are crucial. In situations where similar cross-label samples can be included, the performance on such examples can suggest meaningful learning points that illuminate the limits of the model. The developer can use this to refine the model or articulate this information to potential users. Users should be informed about the data the model was trained on, and the limits of the model, such as which sublabels do not work well. This information is more valuable than simply knowing the model’s overall performance.

## Tip 4: Think about the representativeness of your data

To build an AI for identifying novel cyclin proteins, Mohabatkar and colleagues [23] assembled a training dataset comprising initially 204 non-cyclins and 215 cyclins. Following removal of redundant instances (>90% similarity), they were left with 167 non-cyclins and 166 cyclins. They claimed their classifier achieved approximately 84% accuracy in an evaluation based on cross validation on this dataset. Not to be outdone, other authors working on the same dataset (for example, Yu and colleagues [24]) claimed even higher performance. The models of Mohabatkar and colleagues [23] and Yu and colleagues [24] were impressive with their high reported accuracy. However, the high accuracy they reported regarding their models’ performance may be misleading.

With regard to cyclin proteins, there are about 3,000 cyclin entries in Swiss-Prot [25]. So, the authors could easily have gotten a more convincing cyclin set (they only used 166). Moreover, they would still need to think about a more convincing non-cyclin set. Curating the training data to ensure representativeness has a positive impact on model generalizability.

Although Mohabatkar and colleagues’ [23] dataset is problematic, it does not immediately mean that their model is worthless. It is possible that the model has learnt something useful about cyclins. It might even be possible that whether non-cyclin proteins are appropriately represented is unimportant ultimately to the classifier’s performance. However, without careful curation and means to ensure representativeness, more extensive testing should be called for. For example, we know that most proteins are not cyclins; thus, if the classifier is tested on all protein sequences in a large reference protein data bank (for example, Swiss-Prot) and predicts many sequences, say >5%, as cyclins, the classifier likely has poor specificity.

When applying tip 4, a developer must understand the importance of tapping into prevailing knowledge to design training and test datasets that accurately represent the diversity of real-world instances (**Fig 2D).** Without knowing what the model has learnt to differentiate against, it is hard to mitigate bias and ensure robustness of the model. Users should demand to know to what degree efforts have been undertaken by developers to ensure training data representativeness.

## Tip 5: Consider the impact of batch effects and cross-sample normalization methods

**Fig 3:**
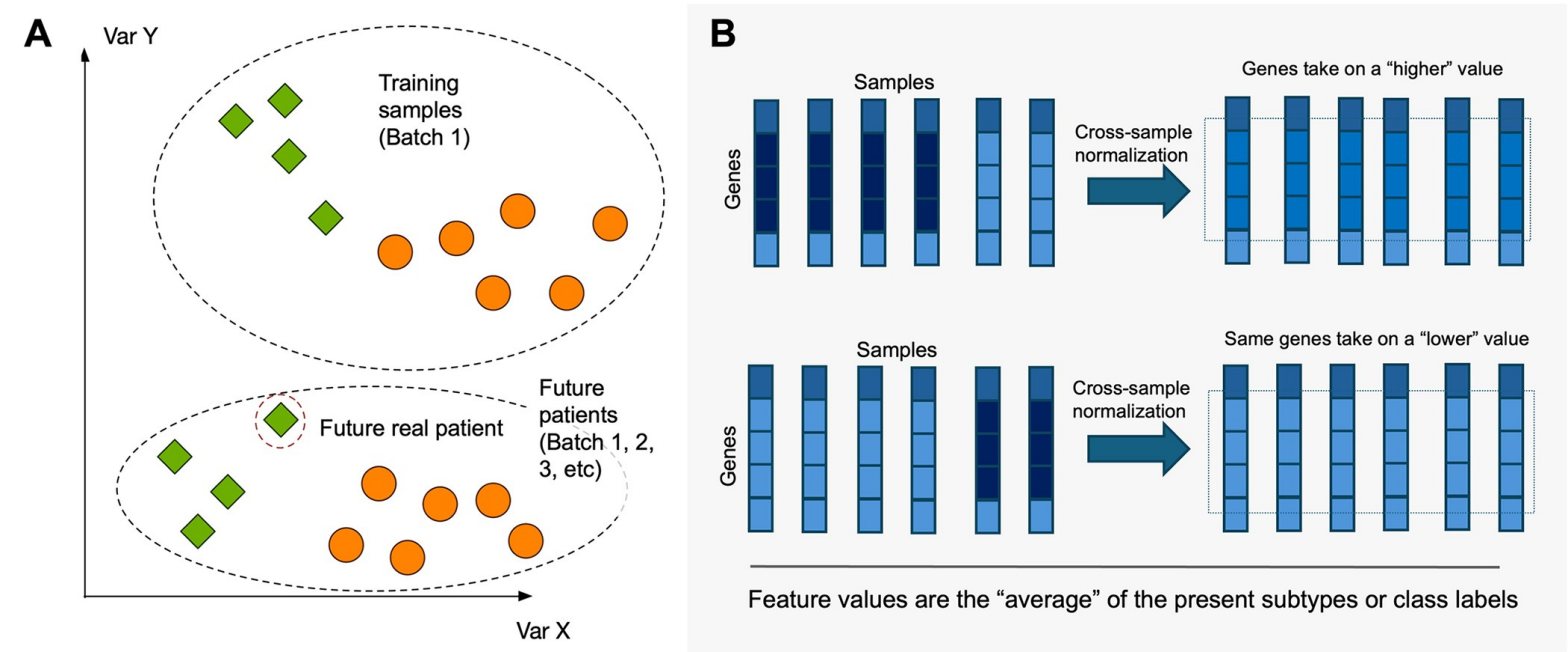
How batch effects (A) and cross-sample normalization (B) can confound ML.

Batch effects are technical biases that arise due to different reagent lot, instrument, or location setting [26–30]. A model that has learnt well during training and performed well on the validation data may fail when deployed because it will be given new samples belonging to new batches that are modulated by other sources of batch-associated technical variations (**Fig 3A**). Since batch effects are inescapable, developers should know that testing the model on new batches is a better practice than merging multiple batches and then splitting the merged dataset into training and test sets (like in a cross validation).

Test set bias describes situations where predictions made by a model for a single sample vary, depending on which other samples the sample is normalized with (**Fig 3B**) [31]. Hence, test-set bias can occur when any cross-sample normalization is performed.

For users, tip 5 is useful for checking model quality. A well-trained model should exhibit robustness and consistency of performance across different batches. In situations where batch effects are overtly dominant (resulting in a completely different distribution), the model should retreat and declare the batch as too different from what it was trained for. A simple way for a user to check the consistency of the model’s performance among different batches is to use a new batch from the user’s side. This helps the user validate the model by comparing this performance with the reported ones. The user should also be concerned with normalization problems such as test-set bias, as this can affect the performance of the model after the model is adopted.

## Tip 6: Do not just rely on your data for feature selection, use prior knowledge

Convergent validation is a procedure for exploiting prior knowledge from related datasets for determining feature quality [32]. In convergent validation, we evaluate the information value of multiple feature sets inferred from *n* datasets and use these to train *n* models. Finally, we challenge these *n* models against 1 validation dataset. The idea for convergent validation was based on the gene signature profiling work of Venet and colleagues [8], where they compared the ability of different breast cancer gene signatures (here, these correspond to feature sets) inferred from different studies against 1 single large high-quality breast cancer dataset, the NKI [33]. Venet and colleagues’ [8] goal was to demonstrate that the prediction performance of each published gene signature is not superior to randomly generated signatures. However, the results can also be taken to mean that each of the signatures has a chance to capture an aspect of what is domain relevant. Thus, some signatures have higher information value than others. Convergent testing can extract the commonalities of successful models, that is, we test all the known signatures on the NKI, determine which ones do well, and then, for this subset of signatures, see what their commonalities are [34]. This procedure is useful for isolating more meaningful explanations in the feature sets.

To apply tip 6, developers should acquire training features from established knowledge and open data repositories and utilize this feature set for model training. Enhancing the feature set’s quality involves employing meta-analysis, mega-analysis, and convergent validation methodologies.

## Tip 7: Calibrate metrics that are affected by class proportion imbalance

Unlike the need for balance in training data, there is no advantage (and no need) for a test set to be balanced. Firstly, a test set that is balanced may deviate greatly from real-world class proportions. Thus, some evaluation metrics (for example, accuracy) that are reported based on such a test set can completely differ from the performance when the model is deployed on real-world populations. That is, the performance reported on an artificially balanced test set must be calibrated and extrapolated in accordance with real-world prevalence before one can say what the expected real-world performance is.

To apply tip 7, developers must calibrate the reported accuracy of a model tested with an artificially balanced dataset in accordance with real-world class-label proportions. This ensures accurate assessment and extrapolation of the model’s performance in real-world scenarios.

For a user, tip 7 is crucial to ensuring consistent assessment of the model’s real-life performance compared to reported results. The user should scrutinize the test set description to identify artificial balancing or deviations from real-world class-label proportions. If these are detected, the user must recalibrate the reported performance in accordance with real-world class-label proportions. Ideally, the user should also generate a test set reflecting natural class-label proportions to validate the performance claimed by developers.

## Tip 8: Benchmark against state-of-the-art tools

Assessing a model’s real-world readiness based solely on accuracy, precision, recall, etc., is inadequate. To establish credibility, it must be compared against cutting edge tools, AI-based or not, for clearer performance evaluation and value-add. Key considerations include the following:

- The selection of tools—this should cover a wide range of different approaches.
- The selection of scenarios—each tool is usually tested and evaluated on a select scenario. The scenario can be biased toward a particular tool, approach, or method. A comprehensive evaluation should also consider potential limitations or biases in the benchmarking scenarios.
- The selection of metrics and factors beyond metrics—besides direct performance metrics, it is important to consider factors such as computational efficiency, scalability, ease of use, and the ability to communicate value and insight to the user.

To apply tip 8, a developer must go beyond reporting high accuracy (or other performance metrics). The developer must also demonstrate superiority against state-of-the-art or prevailing methods in the field. The user should critically assess the real-world value of the approach and compare it against popular state-of-the-art tools. Only then does the model stand a chance of making a meaningful impact.

## Tip 9: Benchmark against null models

**Fig 4:**
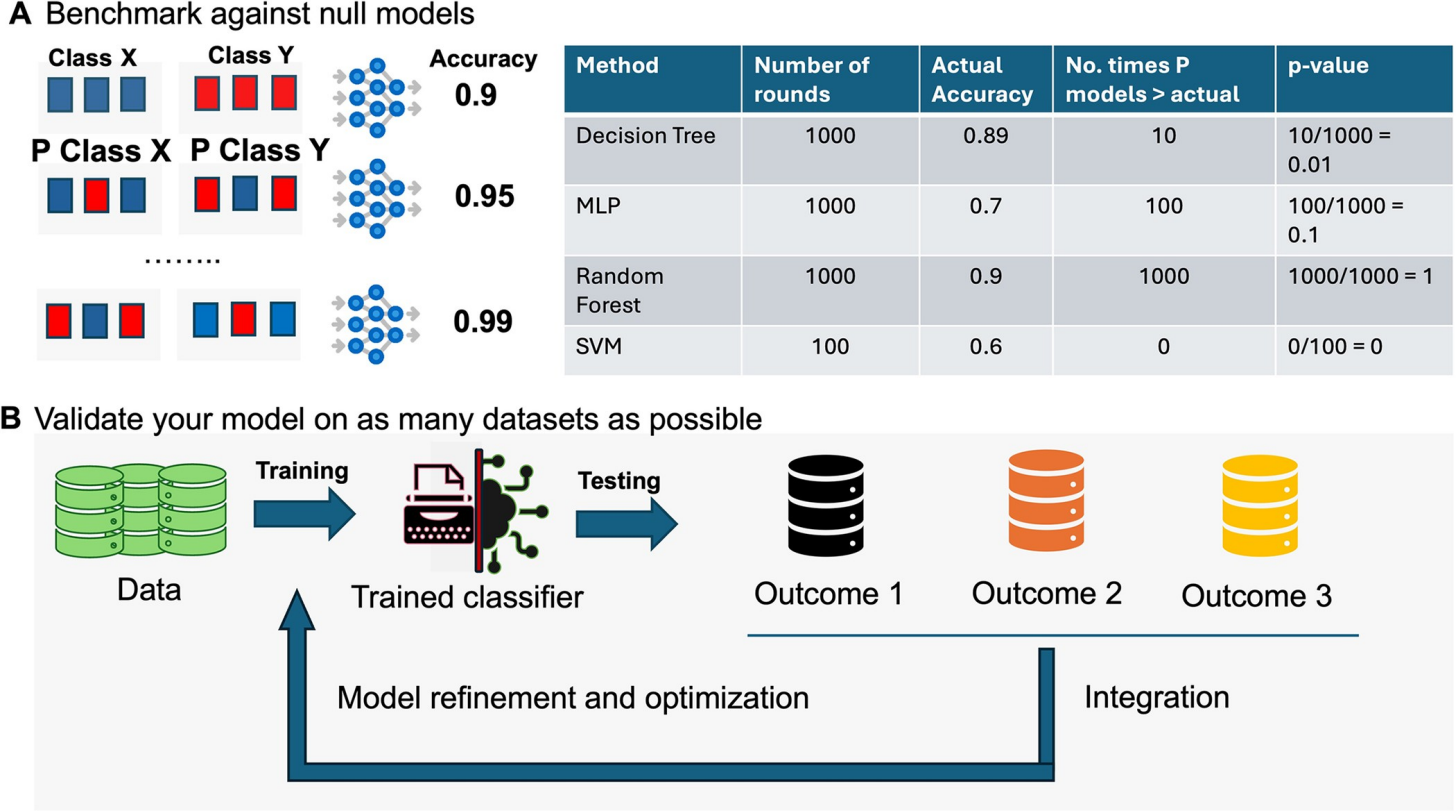
Validation tips covering null model benchmarks (A) and validating the trained model extensively on as many distinct (heterogeneous) dataset as possible (B).

In the study conducted by Venet and colleagues [8], each reported signature’s predictive performance was evaluated by comparing it to randomly generated signatures of the same size. Accuracies from these randomly generated signatures yield an empirical null distribution, which helps in understanding the quality of learning of a model and/or whether the features used are meaningful. For example, if a validation accuracy of 90% was observed for a model, its significance can be determined by comparing it to accuracy of models induced by randomly generated signatures [35] (**Fig 4A**). If only 1 out of 1,000 random signatures equals or exceeds 90%, the significance (*P* value) is 0.001, indicating that the observed accuracy of 90% produced by this feature set is not substitutable by noise. In contrast, if 900 out of 1,000 randomized feature sets perform better, the significance value is 0.90, suggesting the model is using a set of features that is no better than random ones. Including a significance measure to the observed accuracy helps ascertain the utility of the model and the features used.

For tip 9, a developer must not solely rely on the accuracy (or other performance metric) obtained for the model. The developer should also use appropriate null models to ensure that the model’s performance is not attributable to randomness or noise. Applying tip 9 as a user ensures that the model has learned meaningful features for prediction rather than noise. This consideration is essential before adopting the model.

## Tip 10: Validate your model on heterogeneous datasets

To assess a model’s generalizability and robustness, extensive validation using multiple heterogeneous available datasets is essential (**Fig 4B**). Moreover, the findings can be integrated to refine and optimize the model through procedures like hyperparameter tuning and feature selection refinement. This approach also helps uncover issues in the training and validation datasets. For example, persistent failure on a subset of validation data may not indicate generalization failure but could be a representation (tip 4), batch, or normalization-related problem (tip 5). In some cases, it could even be that the comparisons in each dataset differed subtly (tip 3). Hence, by comparing the model against many validation datasets, deeper insights might be gained.

This task can be performed using an approach known as divergent validation [36]. Divergent validation involves a structured approach that includes comparing the model’s generalization to a null model baseline, providing an objective evaluation of its performance across different datasets. By systematically evaluating the model’s success in generalizing to various datasets, researchers can gain valuable insights into its robustness and its ability to adapt to diverse data sources.

When applying tip 10, a developer should use multiple relevant datasets especially during validation to ensure that the model is likely to generalize. Reporting the model’s performance on these datasets will make the model more applicable and appear as a robust model for end users. A user should heed tip 10 when choosing an AI/ML model. It is crucial to opt for a model validated across multiple relevant datasets rather than just one. Otherwise, the model may be overfitted to a specific dataset and perform poorly on other datasets.

## Conclusions

Adopting robust model evaluation techniques for your AI/ML model saves time, energy, and resources but also yields useful insights and enhances trustworthiness. To address the lack of validation frameworks, we propose 10 tips as a starting point toward better model validation practices in biomedical science. Aside from our tips on how to validate model, adopting other best practices including no leakage between training and validation data, open code sharing will help improve research reproducibility, bolster model trustworthiness, and improve adoption of published models.
